# Thyroid hormone resistance resulting from a novel mutation in the THRB gene in a Chinese child: A case report

**DOI:** 10.1097/MD.0000000000033587

**Published:** 2023-04-28

**Authors:** Jinhua Feng, Shuangzhu Lin, Wei Wang, Qiandui Chen, Wanqi Wang, Jiayi Li, Xinyao Wang

**Affiliations:** a Diagnosis and Treatment Center for Children, The Affiliated Hospital of Changchun University of Chinese Medicine, Changchun, Jilin Province, China; b College of Integrated Chinese and Western Medicine, Changchun University of Chinese Medicine, Changchun, Jilin Province, China; c Pediatrics of Traditional Chinese Medicine, College of Traditional Chinese Medicine, Changchun University of Chinese Medicine, Changchun, Jilin Province, China.

**Keywords:** case report, FT3, FT4, growth and development, thyroid hormone resistance

## Abstract

**Introduction:**

Thyroid hormone resistance (RTH) (mim # 188570) is a rare autosomal dominant genetic disorder characterized by reduced thyroid hormone response in target tissues. The clinical manifestations of RTH vary from no symptoms to symptoms of thyroid hormone deficiency to symptoms of thyroid hormone excess.

**Patient concern and clinical findings:**

A 24-month-old girl presented with growth retardation, tachycardia, and persistently elevated thyroid hormones despite antithyroid treatment.

**Diagnosis/Intervention/Outcomes:**

The patient was diagnosed with RTH, after whole exon gene sequencing, found a de novo missense mutation (c.1375T > G,p.Phe459Val) in a novel locus of the thyroid hormone receptor beta gene. She had only mild growth retardation, so the decision was made to monitor her development without intervention. At her last follow-up at 5 years and 8 months of age, she continued to show growth retardation (−2 standard deviation below age-appropriate levels), in addition to delayed language development. Her comprehension ability and heart rate have remained normal.

**Conclusions:**

We report a mild case of RTH caused by a novel thyroid hormone receptor beta gene mutation. RTH should be considered in the differential diagnosis of abnormal serum thyroxine levels during neonatal screening.

## 1. Introduction

Thyroid hormone resistance (RTH) (mim # 188570) is a rare autosomal dominant genetic disorder characterized by a reduced response in target tissues to circulating thyroid hormone.^[1–5]^ First reported in 1967 by Refetoff, DeWind, and DeGroot, RTH has an incidence of 1 in 40,000 to 50,000 with an equal sex preponderance. The genetic rate is approximately 75%.^[1–6]^ The typical biochemical features of RTH are elevated levels of thyroid hormones, serum-free T3 (FT3) and serum-free T4 (FT4), with normal or only mildly elevated levels of thyroid stimulating hormone (TSH). The clinical manifestations of RTH vary from no symptoms to symptoms of thyroid hormone deficiency, such as growth retardation or cognitive impairment, to symptoms of thyroid hormone overdose, such as tachycardia, bone aging, or hyperactivity. Even within the same individual, different target tissues respond to elevated thyroid hormones in an unpredictable manner, with some producing symptoms of hyperthyroidism and others of hypothyroidism. The most common clinical manifestations are goiter and tachycardia.^[7–11]^

To date, >600 cases of RTH have been reported, 85% of which involve pathogenic mutations in the thyroid hormone receptor beta (THRB) gene. The remaining 15% have not been linked definitively to a known mutation, though some studies have shown possible yet unconfirmed gene mutations encoding regulatory co-factors.^[7,12]^

THR is a ligand-dependent transcription factor, located on THR-alpha and THRB genes in humans. As mentioned earlier, mutations in the THRB gene are the most common cause of RTH.^[12]^ The THRB gene is located on chromosome 3p24.2, contains 10 exons, and encodes a protein containing 461 amino acids. This protein consists of 3 functional parts, which correspond to 3 structural domains: an activation domain, a DNA-binding domain, and a ligand-binding domain.^[5,13]^ THRB, mainly located in the nucleus, is widely distributed in various organs such as the liver, kidneys, thyroid, hypothalamus, pituitary gland, and retina.^[14]^ In humans, THRB has 2 subtypes, THRB1 and THRB2, with different biologic actions and distributions throughout the body. THRB1 is mainly expressed in the liver, kidney, thyroid, and other organs. THRB2 is expressed in the hypothalamus, pituitary gland, and retina, both THRB1, and THRB2 have significant effects on the hypothalamic-pituitary-thyroid axis, nerves, and development.^[15,16]^

Approximately 170 different pathogenic mutations in the THRB gene have been reported, among which P. Arg338Trp is the most common.^[5]^ Studies have shown that the mutations mainly involve 2 hotspots of exon 9 and 10, with most contained in 3 clusters rich in CpG for the ligand-binding domain of the corresponding gene.^[17,18]^ THRB heterozygous mutations can result in a mild RTH phenotype, while homozygous deletions can lead to severe generalized RTH symptoms, suggesting that phenotypic severity depends on the number of mutant alleles.^[4,19,20]^ Despite these advances in clarifying the genetic basis of RTH, we do not fully understand the mutational profile of THRB and the phenotype-genotype correlation.

## 2. Case presentation

A 24-month-old girl, born at 36 weeks to a G1P1 mother by cesarean section necessitated by maternal tachycardia, was admitted to our hospital on May 18, 2019, due to “slow growth in height and weight.” Born without a history of asphyxia or need for respiratory rescue, she measured 47 cm long (−2 standard deviation [SD]), weighed 2040 g (−2 SD), had a head circumference of 27 cm (−2 SD), and was breastfed after birth. She was diagnosed with hyperthyroidism at 4 months of age and was treated for 1 month with an unspecified dose of methylimidazole and propranolol (specific drug information is unknown). Unfortunately, thyroid function did not improve. At 6 months of age, the child was 60 cm long (−2 SD) and weighed 4000 g (−2 SD). Despite supplemental food being given starting at 8 months of age, her growth retardation continued, and she measured 69 cm long (−2 SD) and weighed 5000 g (−2 SD) at 12 months.

Apart from growth retardation, she also displayed delayed language development, only able to say “Dad” and “Mom” at 12 months. Her motor development (raised head at 2 months, turned over at 7 months, stood at 10 months, and walked at 12 months) and comprehension level were normal. She had no history of diarrhea or vomiting, had a fair appetite without pica, and had normal stool and urine. She was up to date on vaccinations. As stated earlier, there was no intrauterine hypoxia or birth asphyxia. There was no family history of inherited metabolic or thyroid disease, and both parents were of appropriate height (father and mother, 169 cm and 160 cm tall, respectively).

### 2.1. Physical examination

Upon admission, the child displayed continued growth retardation, measuring 78 cm tall (−2 SD), weighing 8600 g (−2 SD), and with a head circumference of 38 cm. The rest of her physical examination, including skin, head and neck, cardiac, pulmonary, abdominal, lower extremity, motor, and neurologic exam, was normal. Pertinent negatives include lack of skin spots, proptosis, thyroid enlargement or vascular bruits around the gland, cardiac murmurs, or abnormal heart rhythm and rate.

### 2.2. Laboratory and imaging results

Routine blood and urine tests, including liver and kidney function, myocardial enzymes, and genetic metabolism in hematuria, were normal. Thyroid function values are given in Table 1.

**Table 1:** Thyroid function values by date.

	May 15, 2017	May 17, 2017	May 26, 2017	May 31, 2017	June 8, 2017	August 24, 2017	May 18, 2019
FT3	12.01	8.88	18.41	20.06	14.31	16.22	21.37
FT4	83.06	39.28	68.51	73.51	42.34	52.18	64.37
TSH	14.21	3.10	19.50	13.29	17.98	4.83	5.49

Thyroid function test values of the children were calculated according to the time as shown in the table. FT3 = serum free T3, FT4 = serum free T4, TSH = thyroid stimulating hormone.

A thyroid ultrasound was performed, showing thyroid enlargement consistent with goiter (the left lobe measured 8.9 mm × 8.2 mm × 16.7 mm, the right lobe measured 8.5 mm × 8.0 mm × 17.3 mm, and the isthmus was 1.6 mm thick). There was normal blood flow and gland echoes. No associated lymphadenopathy was present.

### 2.3. Results of genetic analysis

**Figure 1.**
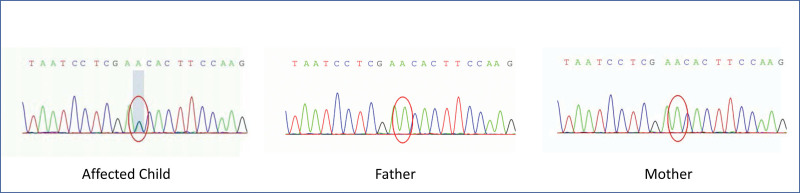
Whole exome gene sequencing results (c.1375T > G).

After obtaining appropriate consent from the child’s family, we performed whole exome gene sequencing on the child and both parents, which found a missense mutation with disease-causing potential in the THRB gene (c.1375T > G, p.Phe459Val) in the child, classified as de novo according to ACMG guidelines since neither parent had the same mutation (Fig. 1).

Based on the THRB gene mutation and mild clinical manifestations, the child was diagnosed with mild extensive RTH.

## 3. Discussion

Thyroid hormone synthesis in humans is controlled by hypothalamic thyrotropin-releasing hormone and pituitary TSH. Thyroid hormones, FT3 and FT4, regulate the production of thyrotropin-releasing hormone and TSH through a negative feedback loop in which thyroid receptor genes are either upregulated or downregulated.^[9,10]^ FT3 is very important for the normal development of the human body. Due to this negative feedback loop, most patients with RTH lead a normal life with normal body shape and development despite high thyroid hormone levels and mild goiter. RTH has a broad phenotypic spectrum and can be divided into generalized RTH and isolated pituitary RTH with peripheral tissue resistance.^[21]^ Generalized RTH is characterized by a lack of response to circulating T3, resulting in negative feedback deficits to FT3 synthesis in the hypothalamic-pituitary-thyroid axis and peripheral tissues, and high levels of FT3. Clinical features of generalized RTH include severe intellectual disability, growth failure, and hyperthyroidism or hypothyroidism.^[17,22–24]^ Some reports describe ADHD in children with RTH, but it typically resolves approximately 1 year after birth.^[25]^ In contrast, isolated pituitary RTH is characterized by localized symptoms, such as those associated with thyroid toxicity.

Clinically, the diagnosis of RTH is somewhat challenging, especially when it coexists with other disorders such as congenital hypothyroidism, congenital hyperthyroidism, or congenital thyroid dysplasia. For instance, some children with RTH may exhibit classic hypothyroid findings such as stunted height and bone age, while also exhibiting classic hyperthyroid findings of the brain and heart,^[26]^ and the processing will be more complicated. In our case, we first suspected congenital hyperthyroidism due to high FT3 and FT4 levels. However, there was continued growth retardation and persistently elevated thyroid hormone levels despite antithyroid treatment. This increased the suspicion of a genetic metabolic disease, which was confirmed with whole exome gene sequencing.

There is currently no agreed-upon and universally effective treatment for RTH. Many patients do not require treatment due to self-regulation by increasing thyroid hormone secretion. Patients with limited thyroid reserve or concurrent autoimmune thyroiditis may benefit from thyroxine therapy, while patients with thyroid hormone excess symptoms may benefit from anxiolytics.^[10,27]^ Other potential therapies, such as thyroid hormone agonists and antagonists, are currently under investigation in animal models.^[28]^

In conjunction with consultation with the child’s parents, we elected to monitor her thyroid levels and clinical status without intervention due to her young age, her poor response to previous drug treatment, and controversial RTH treatment guidelines. At her last follow-up at 5 years and 8 months of age, she continued to have growth retardation (length of 104 cm [−2 SD], weight of 12000 g [−2 SD]), a normal heart rate (120–130 beats per minute), normal comprehension, and continued language difficulty with an inability to express sentences completely. The normal life of the child has not been affected.

## 4. Conclusion

We report a case of RTH with a mild phenotypic profile caused by a THRB gene mutation in a novel locus. Clinicians should consider RTH when serum thyroxine concentrations are elevated in neonatal screening.

## Acknowledgments

We are grateful to the patient and her family for their participation in this study.

## Author contributions

**Conceptualization:** Shuangzhu Lin.

**Resources:** Jinhua Feng.

**Supervision:** Jinhua Feng, Shuangzhu Lin, Wei Wang.

**Writing – original draft:** Qiandui Chen, Wanqi Wang, Jiayi Li, Xinyao Wang.

**Writing – review & editing:** Jiayi Li.
